# Association between the non-high-density lipoprotein cholesterol to high-density lipoprotein cholesterol ratio and metabolic dysfunction-associated steatotic liver disease

**DOI:** 10.3389/fnut.2025.1557751

**Published:** 2025-03-03

**Authors:** Dandan Yang, Hongsheng Dai, Yulu Wang, Jiayi Zhang, Min Wei, Ming Shan, Xiaoqian Zhang

**Affiliations:** ^1^Department of Gastroenterology, Affiliated Hospital of Shandong Second Medical University, Weifang, China; ^2^Clinical Research Center, Affiliated Hospital of Shandong Second Medical University, Weifang, China; ^3^Department of Hepatology, The Fourth Peoples’ Hospital of Huai’an, Huai’an, China; ^4^Department of Ophthalmology, Affiliated Hospital of Shandong Second Medical University, Weifang, China

**Keywords:** MASLD, NHHR, NHANES, dyslipidemia, cross-sectional study

## Abstract

**Background:**

Metabolic dysfunction-associated steatotic liver disease (MASLD) is one of the most widespread chronic liver diseases and a serious global public health problem. Further research to identify novel risk factors associated with MASLD is urgently needed. The non-high-density lipoprotein cholesterol to high-density lipoprotein cholesterol ratio (NHHR) was identified as a novel lipid marker. The objective of this research was to assess the association between NHHR and MASLD in adults.

**Methods:**

This cross-sectional study utilized data from the 2017–2020 National Health and Nutrition Examination Survey (NHANES). MASLD was diagnosed in accordance with controlled attenuation parameter scores and a combination of cardiometabolic risk factors. Multivariate logistic regression analyses, in conjunction with the restricted cubic spline method, were employed to investigate the association between NHHR and MASLD risk. Furthermore, subgroup and interaction analyses were conducted.

**Results:**

This study included 5,269 individuals, with 2,031 individuals diagnosed with MASLD and 3,238 without MASLD. Logistic regression analyses revealed a significant positive correlation between NHHR and MASLD. After the confounding factors were adjusted, each unit rise in NHHR was correlated with a 39% higher probability of MASLD (OR = 1.39, 95% CI: 1.13–1.69). Subgroup and interaction analyses revealed that the positive correlation between NHHR and MASLD held steady regardless of age, gender, race, poverty-to-income ratio, education level, physical activity, body mass index, diabetes, hypertension, dyslipidemia and smoking status (*P* for interaction >0.05). In addition, a non-linear relationship with an S-shaped manner between NHHR and MASLD was found, with an inflection point at 1.59.

**Conclusion:**

Our findings imply that an increasing trend in NHHR is associated with a greater risk of MASLD development. NHHR has the potential to function as an indicator for estimating the likelihood of developing MASLD.

## 1 Introduction

Metabolic dysfunction-associated steatotic liver disease (MASLD), once named non-alcoholic fatty liver disease (NAFLD), represents one of the most widespread chronic liver diseases and constitutes a significant global public health concern (1, 2). MASLD is predominantly distinguished by diffuse hepatocellular steatosis which is engendered by causes apart from alcohol and other identified liver injuries, and it is correlated with an elevated risk of liver-specific complications as well as a variety of extrahepatic diseases (3). Compared with NAFLD, MASLD is defined by more stringent criteria, including hepatic steatosis and at least one indicator of cardiometabolic dysfunction (4). Individuals meeting the diagnostic criteria for MASLD are older and at greater risk of death in contrast to those diagnosed with NAFLD, which may be attributed to cardiometabolic risk factors (4). Approximately 32% of adults in the U.S. are diagnosed with MASLD, indicating its significant prevalence (5). Owing to the epidemics of diabetes and obesity, its prevalence is expected to increase even more (6). Therefore, further research is needed to identify novel risk factors implicated in this disease. This might enable a more comprehensive understanding of the approaches for identifying high-risk individuals and formulating efficient prevention strategies.

Dyslipidemia has been found to be strongly associated with MASLD (7), with abnormal hepatic lipid alterations and dysfunctional lipoprotein metabolism being a predominant factor contributing to the elevated cardiovascular disease (CVD) risk among individuals with MASLD (8). Non-high-density lipoprotein cholesterol (non-HDL-c) encompasses all of the cholesterol that is potentially capable of inducing atherosclerosis and is contained within different types of lipoprotein particles like lipoprotein (a), low-density lipoprotein cholesterol (LDL-c), triglyceride-rich lipoproteins (TRLs), and TRL-remnants (9). Meanwhile, high-density lipoprotein cholesterol (HDL-c), present in the densest lipoprotein particles, protects against atherosclerosis (10). In individuals with MASLD, dyslipidemia typically manifests as heightened concentrations of triglyceride (TG) and LDL-c, along with diminished HDL-c levels, all of which are independently correlated with the incidence and/or mortality of CVD (7). In recent years, the non-HDL-c to HDL-c ratio (NHHR) has been progressively deemed as a novel index for assessing the risk of atherosclerosis and CVD (11, 12). It takes into account two aspects, non-HDL-c and HDL-c, and avoids the drawbacks of previous studies that were exclusive to lipids; moreover, its predictive ability has been shown to outperform that of conventional lipid indicators (13, 14). Studies have revealed that NHHR is not only efficient in evaluating the severity of atherosclerosis, but also shows a substantial correlation and predictive value for a wide variety of diseases. For example, a study carried out by Wang et al. (14) demonstrated that NHHR was significantly correlated with an elevated risk of developing hyperuricemia, accompanied by a U-shaped relationship between them. In addition, an investigation focusing on a sample characteristic of the U.S. adult population highlighted a positive correlation between NHHR and type 2 diabetes, with an 8% increase in the probability of contracting type 2 diabetes for every single-unit rise in NHHR (15). Additionally, emerging investigations have demonstrated that NHHR could be utilized as a predictive indicator for conditions like non-alcoholic steatohepatitis and metabolic syndrome, indicating that it may function as an effective and valuable tool in the prediction of diseases associated with metabolism (16, 17).

Subsequent to the introduction of the new naming system for NAFLD, the connection between the NHHR index and MASLD remains indistinct. Therefore, our study employed the National Health and Nutrition Examination Survey (NHANES) 2017–2020 dataset to conduct a cross-sectional study, aiming to explore the correlation between NHHR and MASLD by means of a more accurate and efficacious methodology.

## 2 Materials and methods

### 2.1 Study population

This research capitalized on the data from the NHANES, which stands as a comprehensive database that sampling the non-institutionalized population across the U.S. biennially. This database extensively investigates the health conditions of U.S. population through questionnaires, laboratory tests, and physical examinations. The research protocol has garnered the endorsement of the Ethics Review Committee of the National Center for Health Statistics, and every subject provided informed consent.

**Figure 1:**
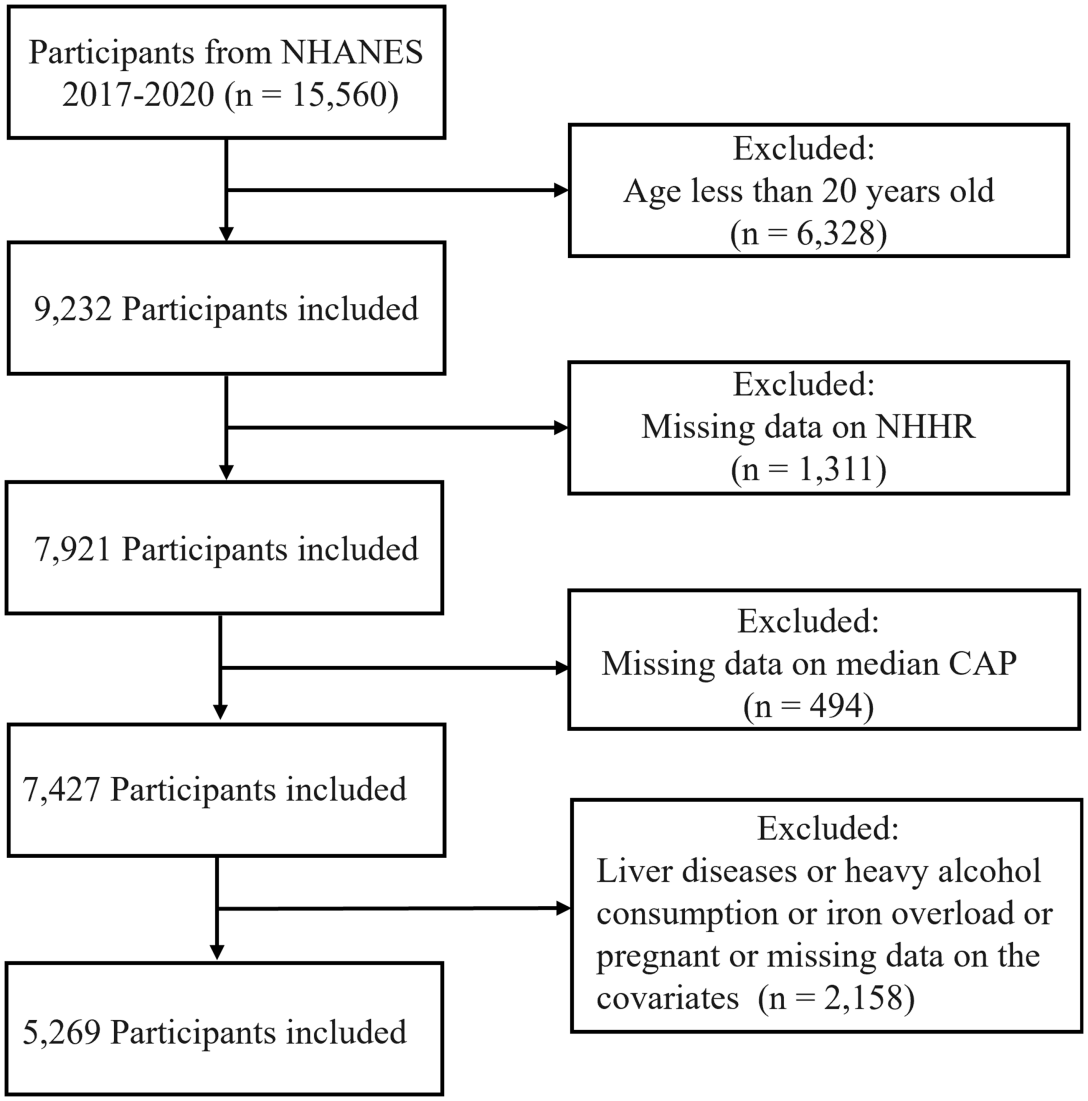
Flowchart of the subject screening process from NHANES 2017 to 2020.

The participants in the present research were selected from the NHANES database from 2017 to 2020, as from 2017, the NHANES first introduced vibration-controlled transient elastography to appraise liver steatosis through the measurement of controlled attenuation parameter (CAP). Initially, 15,560 candidates were included, and the exclusion criteria detailed below were implemented to determine the final study population: (a) age younger than 20 years; (b) insufficient data for the assessment of NHHR; (c) no liver elastography examination; (d) absent data on covariates covering gender, age, race, education level and poverty income ratio (PIR); (e) liver disease or heavy alcohol consumption or iron overload (ferritin ≥1,000 ng/mL); and (f) pregnant. After screening, 5,269 subjects were ultimately included in this research (Figure 1).

### 2.2 Assessment of the NHHR

In this research the NHHR calculated from the lipid levels of the fasting subjects was used as the exposure variable. As previously described in literature (15, 18), NHHR is the ratio of non-HDL-c to HDL-c. Non-HDL-c equals total cholesterol (TC) minus HDL-c. In the subsequent statistical analysis, the NHHR values of all participants in the sample were ranked in ascending order and divided into four groups based on quartile: Q1 (below 1.91), Q2 (between 1.91 and 2.59), Q3 (between 2.59 and 3.48), and Q4 (above 3.48) to facilitate more detailed analysis.

### 2.3 Definition of MASLD

The diagnostic criteria for MASLD was a median CAP score ≥ 285 dB/m and a combination of at least one of the five cardiometabolic risk factors detailed below (19, 20): (a) body mass index (BMI) ≥ 25 kg/m^2^ or waist circumference ≥ 80 cm for females and >94 cm for males; (b) clinically diagnosed diabetes or current use of diabetes management medications or fasting glucose ≥100 mg/dL or 2-h postload glucose levels ≥140 mg/dL or hemoglobin A1c (HbA1c) ≥ 5.7%; (c) blood pressure ≥ 130/85 mmHg or in hypertensive management; (d) plasma TG ≥ 150 mg/dL or on lipid-lowering therapy; and (e) plasma HDL-c ≤ 50 mg/dL for females and ≤40 mg/dL for males.

### 2.4 Covariates

To explore the correlation between NHHR and MASLD, we considered a variety of possible covariates, covering age, gender, race, education level, PIR, physical activity, BMI, smoking status, diabetes, hypertension and dyslipidemia. Information on demographic characteristics was collected through standardized household interviews. In accordance with previous literatures (21, 22), physical activity was categorized as no (no reported leisure-time activity), moderate (activities trigger a rise in heart rate or respiration lasting at least 10 min per week), or vigorous (individuals engaging in high-intensity exercise than described above). For smoking status, never smokers were defined as individuals whose cumulative cigarette consumption was under 100, current smokers referred to those with a smoking history exceeding 100 cigarettes and continued smoking, and former smokers were those who had quit smoking. Data on diabetes, hypertension and dyslipidemia status were obtained from self-report surveys.

### 2.5 Statistical analysis

The NHANES sample weights were incorporated into all analyses to address the intricacies of tiered cluster surveys. Initial normality assessment via the Kolmogorov–Smirnov test confirmed that all continuous variables exhibited non-normal distributions (Supplementary Figure S1). Therefore, continuous variables were presented as weighted medians (interquartile ranges, IQRs), and categorical variables were exhibited as sample sizes along with weighted percentages (%). The distinctions between the non-MASLD and MASLD cohorts were evaluated by chi-square test for categorical variables and Wilcoxon rank-sum test for continuous variables. The relationship between NHHR and MASLD was accessed utilizing multivariable logistic regression models. The variance inflation factor was subsequently used to identify multicollinearity in regression analyses. The NHHR was stratified into quartiles, and individuals in the lowest quartile were categorized into the reference group. In addition, a restricted cubic spline (RCS) regression model was utilized to explore the relationship between NHHR and the incidence of MASLD. Receiver operating characteristic (ROC) curve analysis was employed to evaluate the effectiveness of using TC, non-HDL-c, LDL-c, HDL-c, and NHHR in assessing the risk of MASLD development. Finally, we stratified the subjects according to various covariates and then performed interaction tests to explore whether there was a variation in the relationship between NHHR and MASLD among these subgroups. In this study, all the analytical procedures were carried out with the utilization of R software, and a *p*-value (two-sided) < 0.05 was recognized statistically significant.

## 3 Results

### 3.1 Baseline characteristics

As illustrated in Table 1, this research incorporated totally 5,269 participants with a median age of 48 years, which contained 52.0% female and 48.0% male. Among all participants, 2,031 were identified as MASLD-suspected cases with a median NHHR value of 3.11 (2.38, 3.95), and 3,238 lacked fulfillments of the diagnostic criteria for MASLD with a median NHHR value of 2.30 (1.71, 3.04). All the demographic and clinical traits of both the MASLD cohort and non-MASLD cohort are presented in Table 1. Compared with those in the non-MASLD cohort, individuals in the MASLD cohort tended to be older, male, have lower education levels, less physical activity, and a relatively greater occurrence rate of former smoking habits, and there were more people with diabetes, hypertension and dyslipidemia in the MASLD cohort. Additionally, individuals in the MASLD cohort exhibited increased BMI, CAP, TG, TC and non-HDL-c concentrations, and lower HDL-c levels.

**Table 1:** Baseline characteristics of participants in the NHANES 2017–2020.

Characteristic	Overall *N* = 5,269	Non-MASLD *N* = 3,238	MASLD *N* = 2,031	*p*-value
Age (years), medians (IQR)	48 (34, 62)	46 (32, 61)	53 (39, 64)	<0.001
Age strata, *n* (%)				<0.001
20–39	1,497 (33.1)	1,090 (38.4)	407 (24.4)	
40–59	1,687 (32.7)	948 (30.9)	739 (35.6)	
> = 60	2,085 (34.2)	1,200 (30.7)	885 (40.0)	
Gender, *n* (%)				<0.001
Female	2,758 (52.0)	1,806 (55.4)	952 (46.4)	
Male	2,511 (48.0)	1,432 (44.6)	1,079 (53.6)	
Race, *n* (%)				<0.001
Non-Hispanic White	1,931 (64.8)	1,142 (64.7)	789 (64.9)	
Non-Hispanic Black	1,329 (10.6)	916 (12.0)	413 (8.3)	
Mexican American	626 (8.2)	289 (6.1)	337 (11.6)	
Other Hispanic	528 (7.1)	333 (7.4)	195 (6.5)	
Other Races	855 (9.3)	558 (9.8)	297 (8.7)	
Education, *n* (%)				0.002
More than high school	3,097 (62.3)	1,957 (65.1)	1,140 (57.7)	
High school	1,269 (27.6)	750 (25.2)	519 (31.6)	
Less than high school	901 (10.1)	529 (9.7)	372 (10.7)	
PIR, *n* (%)				0.049
<1.3	1,480 (19.2)	918 (19.2)	562 (19.1)	
1.3–3.5	2,068 (35.6)	1,252 (34.1)	816 (38.1)	
>3.5	1,716 (45.2)	1,065 (46.7)	651 (42.8)	
Physical activity, *n* (%)				<0.001
No	1,527 (23.1)	877 (20.5)	650 (27.3)	
Moderate	1,526 (29.6)	897 (27.7)	629 (32.7)	
Vigorous	2,212 (47.3)	1,460 (51.8)	752 (40.0)	
BMI (kg/m^2^), medians (IQR)	28.9 (25.0, 33.9)	26.5 (23.4, 30.3)	33.7 (29.3, 38.5)	<0.001
Smoke, *n* (%)				0.004
Never	3,124 (59.3)	1,975 (60.4)	1,149 (57.4)	
Former	1,273 (25.4)	689 (22.8)	584 (29.6)	
Current	870 (15.3)	572 (16.7)	298 (13.0)	
Diabetes, *n* (%)	1,155 (16.9)	420 (8.4)	735 (30.9)	<0.001
Hypertension, *n* (%)	2,108 (33.5)	1,070 (25.1)	1,038 (47.4)	<0.001
Dyslipidemia, *n* (%)	2,014 (37.5)	917 (27.3)	1,097 (54.3)	<0.001
CAP (dB/m), medians (IQR)	262 (216, 309)	227 (199, 256)	326 (304, 356)	<0.001
TG (mg/dL), medians (IQR)	90 (61, 138)	76 (53, 113)	122 (87, 170)	<0.001
TC (mg/dL), medians (IQR)	183 (160, 212)	180 (159, 209)	188 (161, 215)	0.012
HDL-c (mg/dL), medians (IQR)	50 (42, 60)	54 (45, 65)	45 (38, 53)	<0.001
LDL-c (mg/dL), medians (IQR)	107 (86, 131)	105 (86, 152)	112 (87, 135)	0.064
Non-HDL-c (mg/dL), medians (IQR)	131 (105, 159)	125 (102, 152)	140 (114, 168)	<0.001
NHHR, medians (IQR)	2.59 (1.91, 3.48)	2.30 (1.71, 3.04)	3.11 (2.38, 3.95)	<0.001

Categorical characteristics were expressed as numbers and survey-weighted percentage, and were compared by chi-square test. Continuous characteristics were expressed as survey-weighted median (interquartile ranges, IQRs), and compared by Wilcoxon rank-sum test.

MASLD, metabolic dysfunction-associated steatotic liver disease; PIR, poverty-to-income ratio; BMI, body mass index; CAP, controlled attenuation parameter; TG, triglyceride; TC, total cholesterol; HDL-c, high-density lipoprotein cholesterol; LDL-c, low-density lipoprotein cholesterol; Non-HDL-c, non-high-density lipoprotein cholesterol; NHHR, non-high-density lipoprotein cholesterol to high-density lipoprotein cholesterol ratio.

### 3.2 Association between quartiles NHHR and MASLD

**Figure 2:**
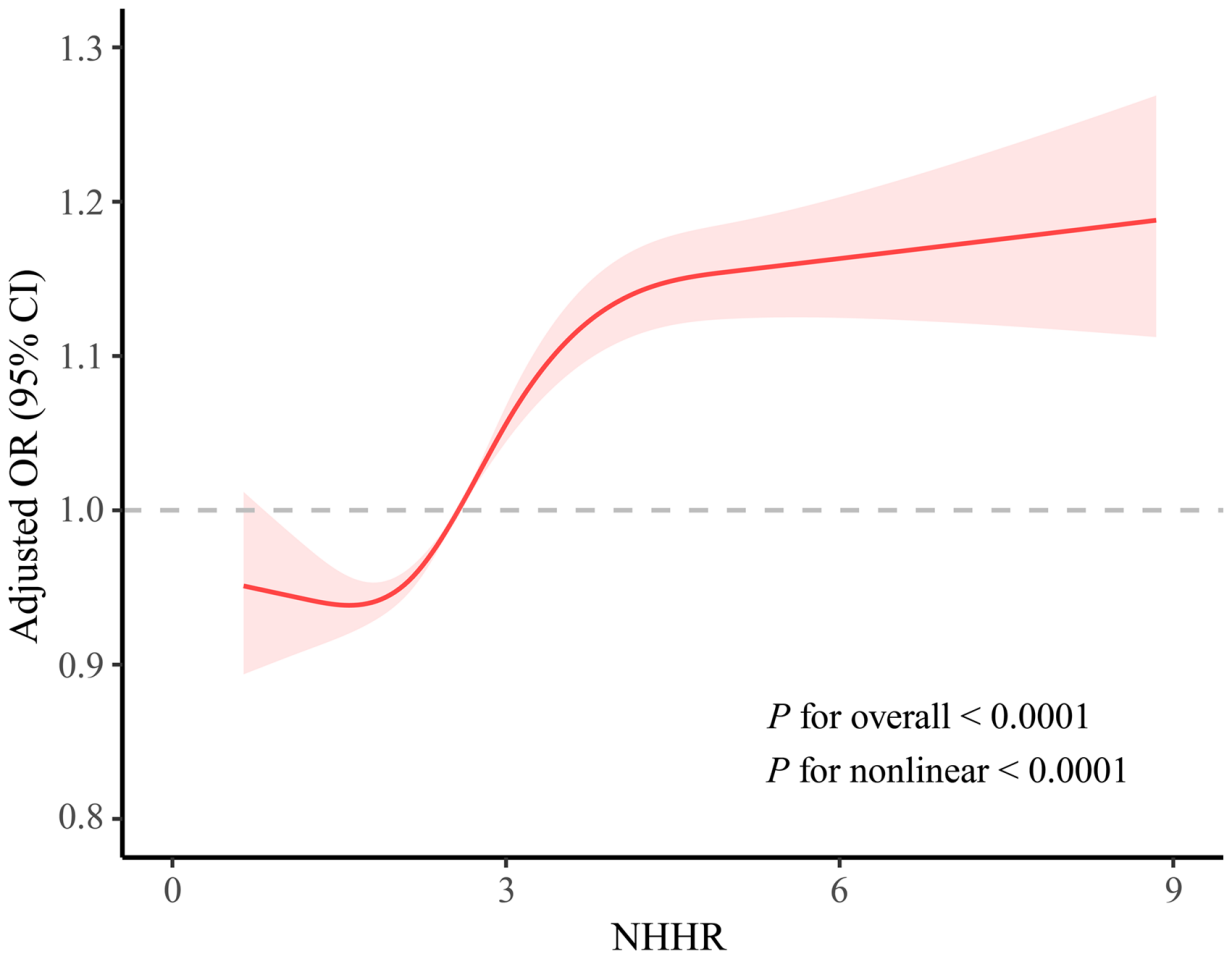
Non-linear relationships between NHHR and MASLD.

**Figure 3:**
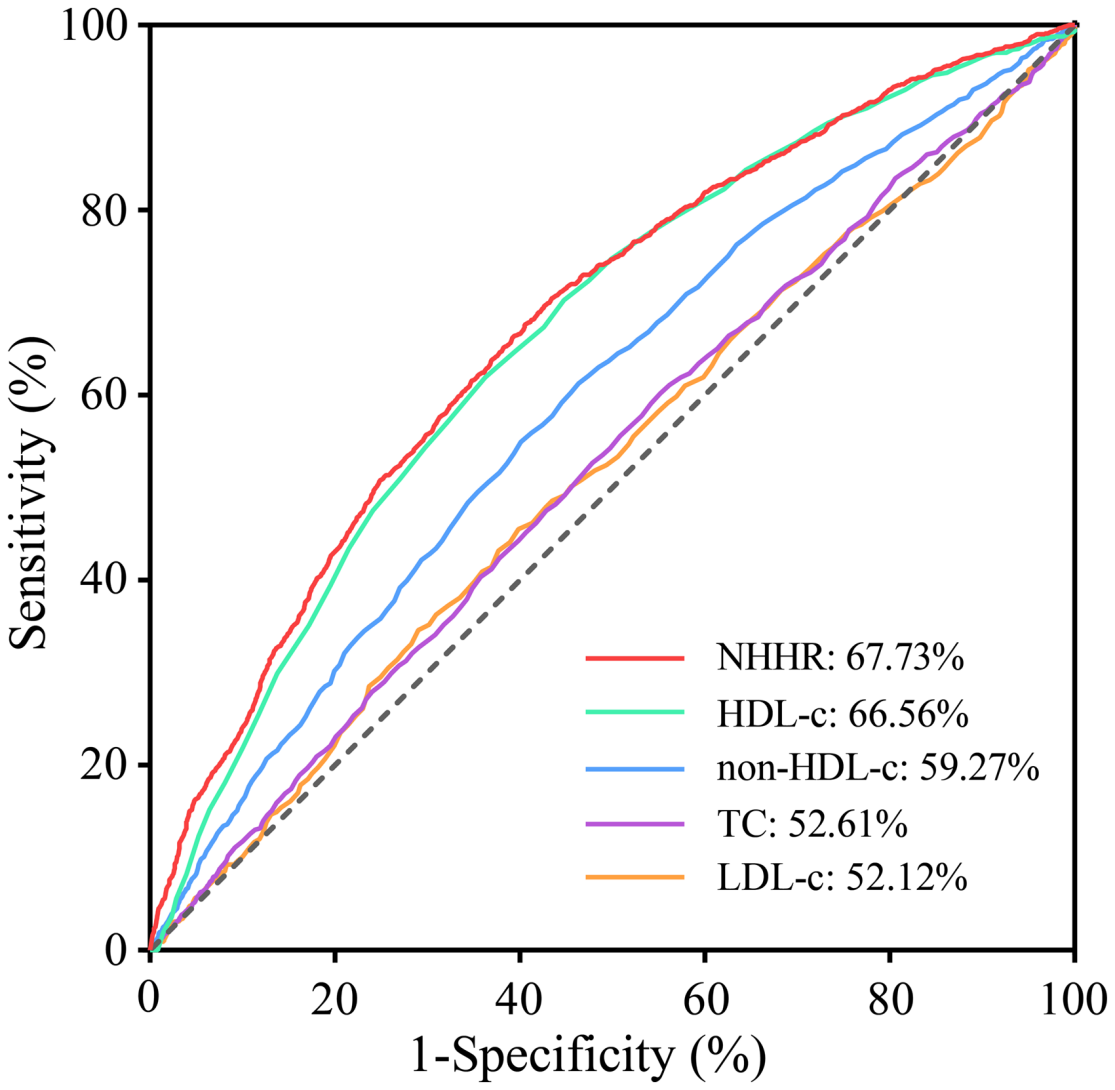
Receiver operating characteristic (ROC) curves for detecting MASLD.

The correlation between NHHR and MASLD is shown in Table 2. When NHHR was regarded as a continuous variable, a positive association was found between it and the likelihood of developing MASLD. This correlative relationship held statistical significance across the logistic regression models without adjustment, with initial adjustment, and with full adjustment. Analysis with an unadjusted model indicated that for every single unit increase in NHHR, there was a corresponding 66% elevation in MASLD risk (OR = 1.66, 95% CI: 1.46–1.89, *p* < 0.001). After adjusting for covariates including age, gender, race, education level, PIR, physical activity, smoking status, BMI, diabetes, and hypertension, an increase of 39% in the prevalence of MASLD was found for each unitary increase in NHHR (OR = 1.39, 95% CI: 1.13–1.69, *p* = 0.006). In the comprehensively calibrated Model 3, in contrast to the subjects in the lowest quartile of NHHR, those in the third quartile (OR = 2.35, 95% CI: 1.55–3.54, *p* = 0.003) and the highest quartile (OR = 3.15, 95% CI: 2.07–4.80, *p* = 0.001) exhibited a notably higher risk of contracting MASLD. As shown in Figure 2, the results of the smoothed curve fitting demonstrate an S-shaped association between NHHR and the probability of contracting MASLD among the population (*P* for overall <0.001). Moreover, there was also evidence of non-linearity between NHHR and MASLD risk, as evidenced by a *p*-value for non-linearity less than 0.001. The correlation between NHHR and MASLD showed an inflection point when NHHR reached 1.59, and to the left of the inflection point, the likelihood of developing MASLD tended to decrease as NHHR increased. According to the results of the ROC curve analysis, the areas under the curve (AUCs) for NHHR, HDL-c, non-HDL-c, TC and LDL-c were 67.73, 66.56, 59.27, 52.61 and 52.12%, respectively (Figure 3).

**Table 2:** The association between NHHR and MASLD (weighted).

	Model 1		Model 2		Model 3	
	OR (95%CI)	*P*-value	OR (95%CI)	*P*-value	OR (95%CI)	*P*-value
NHHR	1.66 (1.46, 1.89)	<0.001	1.65 (1.42, 1.91)	<0.001	1.39 (1.13, 1.69)	0.006
NHHR quartile						
Quartile 1	1.00 – reference	–	1.00 – reference	–	1.00 – reference	–
Quartile 2	1.72 (1.31, 2.26)	<0.001	1.72 (1.26, 2.34)	0.003	1.29 (0.78, 2.12)	0.249
Quartile 3	3.54 (2.82, 4.45)	<0.001	3.40 (2.67, 4.33)	<0.001	2.35 (1.55, 3.54)	0.003
Quartile 4	5.49 (4.37, 6.89)	<0.001	5.48 (4.24, 7.09)	<0.001	3.15 (2.07, 4.80)	0.001
*P* for trend		<0.001		<0.001		0.004

Model 1: no covariates were adjusted.

Model 2: adjusted for age, gender, race, education level and PIR.

Model 3: further adjusted for physical activity, smoking status, BMI, diabetes and hypertension.

### 3.3 Subgroup analysis

**Figure 4:**
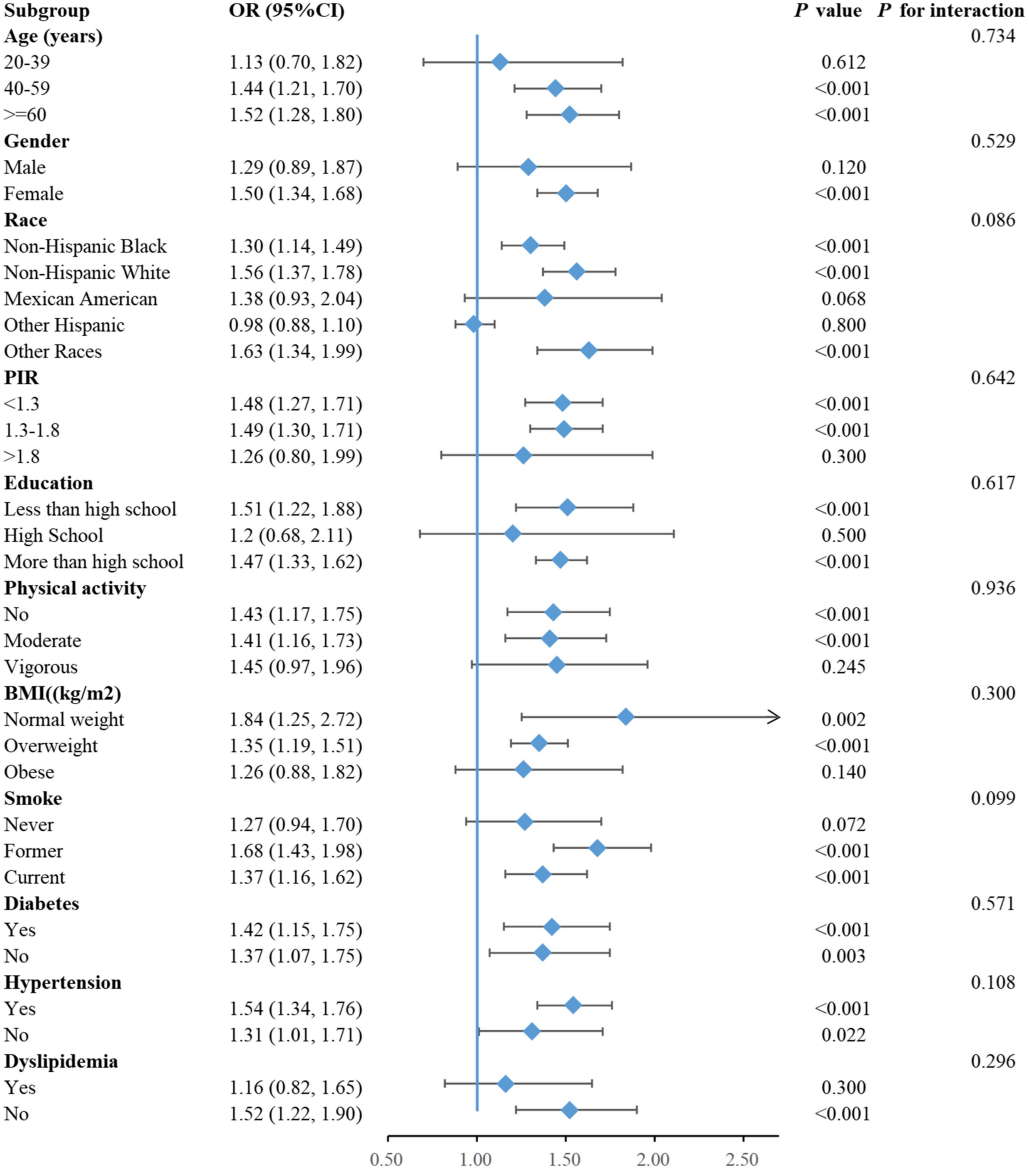
Forest plot of the associations of NHHR with MASLD.

Taking into account all factors, subgroup analyses were performed to assess the reliability of the relationship between NHHR and MASLD risk within diverse populations, and the results are illustrated in Figure 4. No significant interactions were detected among age, gender, race, PIR, education level, physical activity, BMI, smoking status, diabetes, hypertension and dyslipidemia, indicating that the positive correlation between NHHR and MASLD remained unaffected by these variables (*P* for interaction >0.05). Among these, strong association was identified in participants aged 40–59 years (OR = 1.44, 95% CI: 1.21–1.70) as well as those older than 60 years (OR = 1.52, 95% CI: 1.28–1.80). Notably significant correlations were also detected among Non-Hispanic Black individuals OR = 1.30, 95% CI: 1.14–1.49), Non-Hispanic White individuals (OR = 1.56, 95% CI: 1.37–1.78), and other races (OR = 1.63, 95% CI: 1.34–1.99). Individuals with lower-income (OR = 1.48, 95% CI: 1.27–1.71) and middle-income (OR = 1.49, 95% CI: 1.30–1.71) levels demonstrated meaningful correlations. Lifestyles of no daily activity (OR = 1.43, 95% CI: 1.17–1.75) and moderate physical activity (OR = 1.41, 95% CI: 1.16–1.73) also exhibited significant associations. In addition, significant correlations were also shown among those who had normal weight (OR = 1.84, 95% CI: 1.25–2.72), were overweight (OR = 1.35, 95% CI: 1.19–1.51), were currently smoking (OR = 1.37, 95% CI: 1.16–1.62), had a smoking history (OR = 1.68, 95% CI: 1.43–1.98), or had no dyslipidemia (OR = 1.52, 95% CI: 1.22–1.90), with *p*-values <0.05 for all associations.

## 4 Discussion

The present study based on U.S. adult population investigated the association between NHHR and the probability of MASLD development. The present research demonstrated that individuals without MASLD possessed a considerably lower NHHR values than did those diagnosed with MASLD. Moreover, multivariable regression analysis revealed that elevated NHHR levels were strongly correlated with a higher risk of MASLD. Additionally, the relationship between NHHR and MASLD exhibited a non-linear and S-shaped profile, with an inflection point at 1.59.

The NAFLD is correlated with an increased risk of intra- and extra-hepatic malignancies and constitutes the primary etiological factor for liver-related mortality worldwide (23). Recently, MASLD has gradually supplanted NAFLD on account of the increasing prevalence of metabolic syndrome and obesity, emphasizing the importance of cardiometabolic risk factors (24). MASLD represents a multisystem disorder arising from systemic insulin resistance, dyslipidemia, and associated metabolic dysfunction (3). Patients diagnosed with MASLD manifest hepatic lipid metabolism imbalance characterized by increased lipolysis within adipose tissue, high hepatic *de novo* lipogenesis, impaired lipid oxidation and alterations in very-low-density lipoprotein secretion, which ultimately leads to hepatic lipid accumulation (25). The accumulation of lipids within hepatocytes generally induces lipotoxicity, subsequently resulting in chronic inflammation in hepatic tissue by mediating organelle dysfunction, endoplasmic reticulum stress, oxidative stress, ferroptosis, and the activation of proinflammatory factors, further aggravating insulin resistance (26, 27). In addition, the accumulation of toxic lipid metabolites triggers hepatocellular stress, damage and death, consequently leading to fibrogenesis and DNA mutations that eventually increase susceptibility to the progression of cirrhosis and hepatocarcinoma (28, 29).

In previous studies, LDL-c has been regarded as the central target of lipid metabolism disease management, whereas non-HDL-c, which encompasses the entirety of plasma lipoprotein particles apart from HDL-c, has been recognized as an independent risk factor and prognostic indicator of CVD (30–33). Notably, the association between dyslipidemia and liver diseases has been extensively explored (8, 34). A longitudinal cohort study conducted among Japanese adults demonstrated that LDL-c and non-HDL-c showed a significant positive correlation with the risk of developing NAFLD, while HDL-c was negatively associated with NAFLD risk (35). Furthermore, a prospective cohort study encompassing 147 individuals free of liver disease with a 7-year follow-up time revealed that non-HDL-c served as an independent prognostic indicator for the initial occurrence of NAFLD, and its predictive effect was stronger than that of other lipid components, such as TC, LDL-c and HDL-c (36). This association may result from the hepatotoxicity of non-HDL-c and account for the link between CVD and NAFLD. In addition, non-HDL-c has also been demonstrated to be associated with the severity of liver disease, and among subjects not taking lipid-lowering medications, the levels of non-HDL-c in patients with non-alcoholic steatohepatitis are markedly higher than those in patients with steatosis (37). In addition, serum remnant cholesterol, one of the constituents of non-HDL-c, was also verified to be positively correlated with the risk of NAFLD, and its predictive capacity for the progression of NAFLD surpassed that of traditional lipid components (38). This correlation was significant among individuals who exhibited normal levels of HDL-c, LDL-c, and triglycerides (38).

NHHR, a newly-emerged comprehensive marker of blood lipids proposed in recent years, encompasses all pertinent information regarding anti- and pro-atherosclerotic lipid particles, reflects the equilibrium among lipoproteins, and has progressively emerged as a potential marker for metabolic disorders (15, 39). A cross-sectional study encompassing 30,482 U.S. adults demonstrated that increased NHHR levels were significantly associated with a heightened gout prevalence. After accounting for various confounding factors, such as age, gender, race, education level, and so on, it was found that for every single unit increase in NHHR, the probability of gout incidence increased by 10% (40). Furthermore, a study including 12,578 U.S. adult subjects with prediabetes or diabetes disclosed that NHHR exhibited an L-shaped connection with cardiovascular mortality and a U-shaped correlation with all-cause mortality, with inflection points of 2.83 for cardiovascular mortality and 2.72 for all-cause mortality, respectively (41). Specifically, once baseline NHHR surpassed the inflection point, NHHR was positively associated with cardiovascular and all-cause mortality among patients with prediabetes or diabetes (41). However, studies on the correlation between NHHR and the risk of developing MASLD are currently limited, so it is unclear whether NHHR can be utilized as a valid marker to predict MASLD. The present study revealed that NHHR has a greater predictive value for MASLD with an AUC of 67.73% than traditional lipid related indicators, such as TC, non-HDL-c, LDL-c and HDL-c. Similar results were also reported in studies investigating the predictive effects of NHHR and conventional lipid parameters on hyperuricemia and diabetes. A study exploring the relationship between NHHR and the prevalence of hyperuricemia in US adults revealed that compared with non-HDL and HDL-c, NHHR had a better predictive effect on hyperuricemia, with an AUC of 61.76% (14). Similarly, compared to other conventional lipid parameters, NHHR is a more effective predictor of diabetes risk and an independent risk factor for new-onset diabetes mellitus in the general population (42).

After adjustment for potential confounders other than dyslipidemia, we found that each one-unit increase in NHHR was associated with a 39% increase in the risk of developing MASLD. A previous study exploring the relationship between lipid parameters and NAFLD risk, after adjusting for potential confounders other than BMI, reported that every single unit increase in TC was accompanied by a 17% increase in NAFLD risk, every single unit increment in LDL-c was corresponded to a 29% elevation in NAFLD risk, and every single unit rise in non-HDL-c was correlated with a 36% augmentation in NAFLD risk (35). In addition, RCS regression model in this study revealed a non-linear S-shaped relationship between NHHR and MASLD risk, which was consistent with prior research exploring the relationship between NHHR and the risk of diabetes development (42). This study bridges the gaps of prior research and expands the application of lipid ratios, indicating that NHHR could serve as a predictive indicator for MASLD. Notably, the correlation between NHHR and NAFLD has also been explored in previous studies. Huang et al. demonstrated increased NHHR levels are in close correlation with an augmented risk of CAP-defined NAFLD (43). However, Huang et al. (43) did not consider cardiometabolic risk factors, which served as potentially confounding factors and significant parts of defining MASLD. In the current research, MASLD was identified according to the existence of hepatic steatosis in combination with one or more of the five cardiometabolic risk factors, thus overcoming the limitations of the research conducted by Huang et al. (43).

This research has several strengths. First, this was a large sample size population-based study, providing evidence of the correlation between NHHR and MASLD. Second, the adjustment of covariates and subgroup analysis deepened the finding of the association between NHHR and MASLD. However, some limitations should be addressed in this study. First, we could not reveal cause-effect relationship between NHHR and MASLD in our study since it was a cross-sectional design. Second, liver biopsy is a better standard clinical test than CAP value from transient elastography we measured in this study for the diagnosis of liver steatosis. Third, the patients with MASLD enrolled in this research were all obtained from the U.S. population in NHANES database, and the extrapolation of the findings to other populations demands further exploration. Therefore, more prospective investigations are essential to further validate and explore the correlation between NHHR and MASLD.

## 5 Conclusion

Our findings imply that a heightened NHHR is significantly correlated with a greater propensity for developing MASLD in U.S. adults. This implies that lipid management and the possible influence of NHHR on MASLD should be considered during the prevention, evaluation, and treatment procedures for this disease. It is necessary to conduct more high-quality research in the future to validate our findings.

## Data Availability

Publicly available datasets were analyzed in this study. This data can be found at: the NHANES repository (https://www.cdc.gov/nchs/nhanes/).
