# Supplementary material for: Association between the non-high-density lipoprotein cholesterol to high-density lipoprotein cholesterol ratio and metabolic dysfunction-associated steatotic liver disease
Source: Front Nutr. 2025 Mar 3;12:1557751. doi: 10.3389/fnut.2025.1557751 (PMC11912010; doi:10.3389/fnut.2025.1557751)

Figure S1. **The distribution of the continuous variables in this study.** NHHR, non-high-density lipoprotein cholesterol to high-density lipoprotein cholesterol ratio; BMI, body mass index; CAP, controlled attenuation parameter; TG, triglyceride; TC, total cholesterol; HDL-c, high-density lipoprotein cholesterol; LDL-c, low-density lipoprotein cholesterol; non-HDL-c, non-high-density lipoprotein cholesterol;

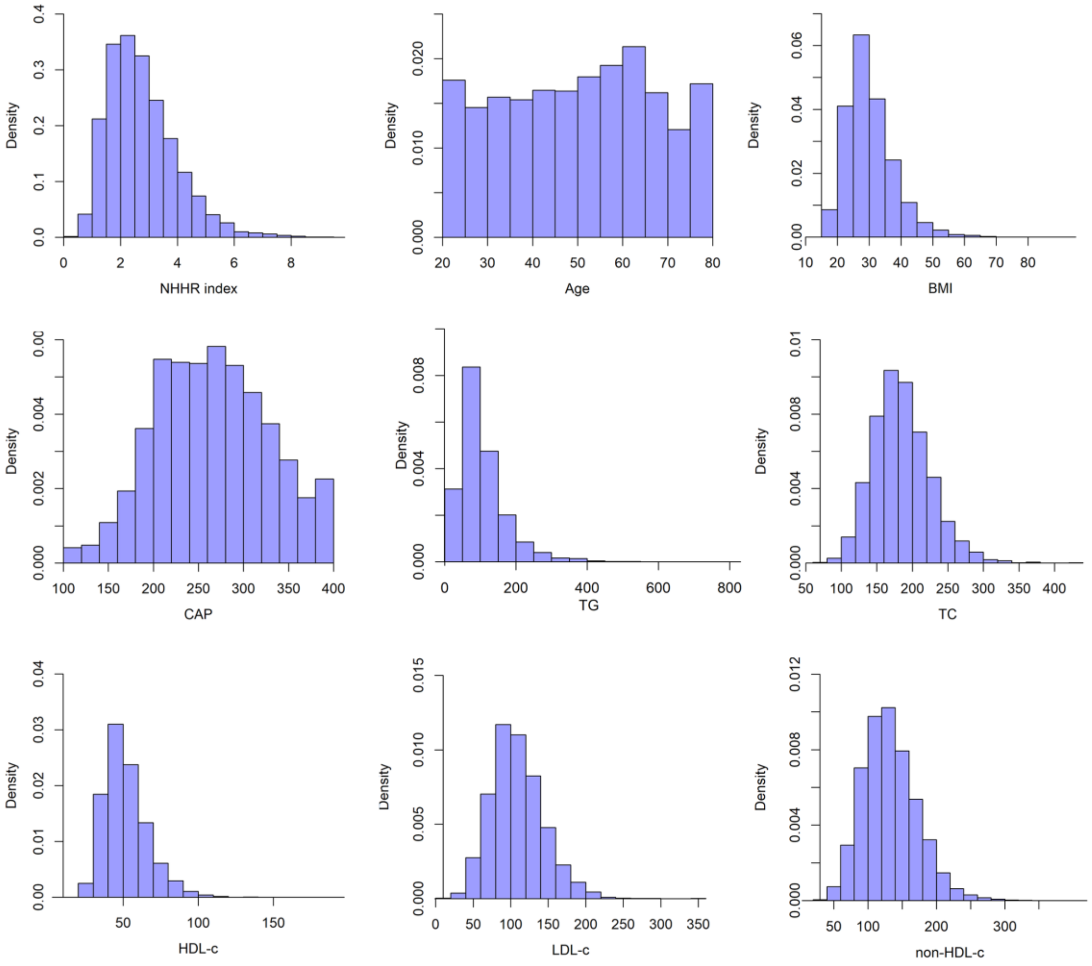

Supplement: Supplementary file 1 [file Image_1.PDF]
